# Combined application of vermicompost and mineral K fertilizer improves root yield of sweet potato [*Ipomoea batatas* (L.) Lam] in Southern Ethiopia

**DOI:** 10.1016/j.heliyon.2025.e42250

**Published:** 2025-01-24

**Authors:** Alemu Degwale, Geletaw Kebede, Ashenafi Woldeselassie, Fanuel Laekemariam

**Affiliations:** a University of Gondar, College of Agriculture and Environmental Sciences, Ethiopia; b Wolaita Sodo University, College of Agriculture, Ethiopia

**Keywords:** Dry matter content, K, Marketable yield, Root dry mass, Total root yield

## Abstract

The high cost of mineral fertilizers, lack of credit, and delivery delays constrain crop production in the developing world like Ethiopia. Farmers apply little or no fertilizer to sweet potatoes leading to lower root yield (8 t ha^−1^) compared with 60 t ha^−1^ in research fields. Eventually, this has led to a decline in food security. Although the rate depends on many factors, organic inputs such as vermicomposts are suggested as alternatives or combined with mineral fertilizers. This research was therefore initiated to investigate the effect of the combined application of vermicompost (VC) and mineral K fertilizer on the root yield of sweet potatoes. Treatments were 0, 4, 8, or 12 t ha^−1^ VC and 0, 50, 100, or 150 kg ha^−1^ K laid out in a Randomized Complete Block Design, factorial arrangement with three replications. The combined application of 12 t VC ha^−1^ with 150 kg K ha^−1^ produced the highest total root yield (57.35 t ha^−1^), marketable root yield (46.45 t ha^−1^), and harvest index (16.5 %). These values were comparable to those of 8 t VC ha^−1^ and 150 kg K ha^−1^. Dry matter content was increased by 35.5 % in location-1 and 38.2 % in location-2 at combined rates of 12 t VC ha^−1^ and 150 kg K ha^−1^ that did not differ from 8 t VC ha^−1^ and 150 kg K ha^−1^. Root diameter, dry matter content, harvest index, marketable, and total root yield had a strong positive relationship. Applying 8 t VC ha^−1^ with 150 kg K can improve root yield aboveground biomass of sweet potato.

## 1 Introduction

Sweet potato [*Ipomoea batatas* (L.) Lam] belongs to the family *Convolvulaceae*, is originated from Central America, and is now widely grown in almost all soil types of tropical, subtropical and warm temperate regions. It ranks as the world's fifth-most significant food crop, second among root and tuber crops only to the Irish potato, and first in terms of yielding edible energy per hectare per day. Sweet potato produces storage roots rich in carbohydrates, vitamins A, B complex, C, E, and minerals such as potassium, calcium and iron [1,2]. Its leaves are used as a vegetable; storage roots are boiled, fried, baked, roasted, or fermented into food and beverages [3]. Sweet potato storage roots are processed into flour for bread making, starch for raw material in industrial starch, and alcohol. The flour is used in sweetening and fortifying baby foods [4]. Sweet potato is cultivated year-round withstanding harsh conditions such as drought. Due to the ability of on-farm storage, sweet potatoes are harvested for daily intake. Hence, it is thought of as a typical food security crop for developing countries and known as “an insurance crop” [5].

Sweet potato is considered a crop well suited to soils that are often infertile, and root yield is sometimes depressed in very fertile or heavily fertilized soils [6]. Nevertheless, it requires fertile, well-drained, sandy loam soil, moderate to slightly acidic with a pH of 5.6–6.5 [[7], [8], [9], [10]]. Since sweet potato is a high carbohydrate producer, it requires an optimum supply of nutrients with a high demand for organic matter, N and K to maintain photosynthesis and carbohydrate assimilation [[11], [12], [13]].

Vermicomposting is a simple, eco-friendly process that turns organic wastes into a greater variety of organic fertilizers while sequestering CO_2_ [14]. Vermicompost (VC) is the worms’ casting; a highly nutritive organic fertilizer and powerful growth promoter and among the strategy of organic carbon buildup in the soil [[15], [16], [17], [18]]. Earthworms along with associated microbes facilitate the decomposition of organic materials and make nutrients available to plants [19]. Vermicompost has been recognized as an effective means for improving soil aggregation, structure and fertility, moisture-holding capacity, and Cation Exchange Capacity (CEC) [14]. It is a soil fertility booster that restores its natural soil properties against destructive chemical fertilizers. Vermicompost (VC) contains enzymes and natural growth stimulants that, along with nutrients and humic acid, promote plant growth and yield [20,21]. It also promotes beneficial microbes and soil biodiversity which in turn enhances plant growth directly by production of plant growth-regulating hormones and enzymes and indirectly by controlling plant pathogens [[22], [23], [24], [25]]. Due to its innate biological, biochemical and physicochemical properties, VC is used to promote sustainable and safe management of agricultural, industrial, and domestic wastes.

Potassium (K) and Nitrogen (N) are the elements removed by sweet potatoes in larger quantities than others [26]. Leaves, vines, stems and tubers usually remove a substantial quantity of K from the soil. A 50 t ha^−1^ root output of sweet potatoes removes roughly 250 kg ha^−1^ K by its roots and 126 kg ha^−1^ by its vines, but the quantity of N and P removed is 110 kg ha^−1^ and 25 kg ha^−1^ by its roots, and 105 kg ha^−1^ and 13 kg ha^−1^ by its vine, respectively [27]. Potassium is involved in vital physiological processes such as carbohydrate synthesis, translocation of photosynthates, protein synthesis, control of ionic balance, regulation of plant stomata, turgor maintenance, stress tolerance, water use efficiency, and activation of plant enzymes [[28], [29], [30], [31]]. Potassium in soil solutions has a high risk of leaching and is removed from the root system. Hence the application of mineral K fertilizers is suggested to enhance the productivity of sweet potatoes [32]. Liu et al. [33] confirmed that the K requirement of sweet potato is highly dependent on soil types, soil moisture content, soil inherent K, and other nutrient composition.

In Ethiopia, sweet potato productivity in farmers' fields averages just 8 t ha^−1^ [34], which is considerably lower than the potential yield of 50–60 t ha^−1^ obtained in research settings [35]. This higher yield is achieved through optimized agronomic practices, such as applying irrigation water to maintain field capacity weekly, balanced NP fertilizer application (including 20 t ha^−1^ of farmyard manure and 180 kg ha^−1^ of phosphorus), as well as the use of appropriate planting materials, effective planting methods, and suitable cropping systems [35]. This highlights a significant disparity in sweet potato yields between farmers' fields and research plots in Ethiopia. Several factors may contribute to this difference, including low fertility, soil acidity [36,37], inadequate planting materials, inappropriate agronomic practices, recurrent drought, and pest infestations. In Southern Ethiopia, soil fertility has declined due to continuous cultivation, the removal of crop residues, overgrazing, insufficient fertilizer use, and poor soil management practices [[38], [39], [40]]. Soil organic carbon (OC), total N, available P, exchangeable K, S and micronutrients (B, Cu, Fe and Zn) are low [41]. The soil is very strongly acidic, with a pH of 5.30 (H_2_O), which may hinder the growth and root development of sweet potatoes in the study areas [42]. On the other hand, farmers in Southern Ethiopia cultivate sweet potatoes with little or no application of any fertilizer. This has resulted in low crop yields leading to reduced food security and increased poverty.

Most smallholder farmers in the study area appreciate the value of inorganic fertilizers, but they are seldom able to apply at an appropriate time and recommended rates because of high cost, lack of credit, delivery delay, and low and variable returns [38]. Organic inputs such as vermicompost (VC) are often proposed as alternatives or as a combined application with mineral fertilizers. The application of VC along with mineral K fertilizer could improve the availability of N, P, K, micronutrients and soil microbes in the rhizosphere [43] and as a result increase nutrient uptake, plant metabolism, photo-assimilates and enhance tuber qualities. However, information are scanty on the effect of combined application of VC with mineral K fertilizer on the growth and root yield of sweet potatoes in the study areas. Hence, this research was initiated to investigate the effect of the combined application of vermicompost and mineral K fertilizer on the root yield of sweet potatoes.

## 2 Materials and methods

### 2.1 Description of the study area

A field experiment was conducted in the main rainy season of 2018/19 at Sodo zuria (location-1) and Boloso sore (location-2) districts of Wolaita zone, Southern Ethiopia, located at 6°49′ N of latitude and 39°47’ E of longitude, about 370 km southwest of Addis Ababa. The zone is situated at an altitude of about 2200 m a.s.l. The soils are highly weathered, well-drained, deep, highly leached, and acidic with low organic carbon, nitrogen and phosphorus contents [44]. The average monthly temperature ranges between 17.9 °C (July) and 22.0 °C (March) with a mean annual temperature of 19.8 °C. There is a bimodal rainfall (February to April and June to September) of 1489 mm a year. The textural class and some chemical characteristics of the experimental soils are presented in Table 1.

**Table 1:** Physicochemical properties of the experimental soils before planting and the vermicompost (VC).

Parameter	location-1		location-2		VC		
Clay (%)	63.4		59.2		–		
Silt (%)	38.6		28.2		–		
Sand (%)	11.5		14.7		–		

**Textural class**	**Clayey**		**Clayey**		**-**		
		Description		Description		Description	Reference

pH_H2O_	6.0	Moderately acidic	5.8	Moderately acidic	7.1	Neutral	[45]
Total N (%)	0.18	Low	0.2	Low	1.78	Very high	[46]
Available P (mg kg^−1^)	16.6	Medium	14.2	Low	22.1	Medium	[47]
Extractable K (mg kg^−1^)	88	Very low	72	Very low	102	Low	[47]
OC (%)	2.48	Low	2.4	Low	32	Very high	[46]

### 2.2 Description of the experimental material

**The planting material:** A standard variety of sweet potatoes named Awassa-83 was used for the experiment by collecting vine cuttings from Hawassa Research Center. The cultivar was selected according to agroecological adaptability, disease resistance, and high yield potential.

### 2.3 Fertilizer material

Vermicompost and potassium chloride (KCl) (60 % K) were used as sources of organic and potassium fertilizers, respectively. Vermicompost was prepared in a 1.5 m × 2 m area bin. It was prepared at the demonstration site of the Department of Horticulture consisting of 30 cm layer of enset (*Ensete ventricosum* L.) leaves, 10 cm layer of animal dung, 10 cm layer of ash, 30 cm layer of green leaves of weeds, and 30 cm layer cafeteria wastes (vegetable residues, dry food scraps i.e. bread, *Injera,* e.t.c). Shade was provided over the vermicompost bin with plastic sheets to prevent the entry of rainwater and exposure to direct sunshine. The earthworm *Eisenia fetida*, collected from Amhara Regional Agricultural Research Institution (ARARI), was introduced as decomposition commences (2 weeks later) at a rate of 100–200 worms m^−2^. To keep birds, moles, and shrews away from the earthworms, the bins were covered with grasses and wire mesh. The bedding material was watered every three days to maintain about 60 % moisture. This could make it easier for the worms to move around, stretch their skin to enable breathing through it, as well as cool down the rising temperature during decomposition. After nearly 90 % of the garbage had broken down and turned into soil, the water was turned off. Maturity was judged visually by observing the formation of the granular structure of the vermicompost at the surface of the bin [48]. After two months, organic refuse changed into a soft, spongy, odorless dark brown compost ready for harvesting. By exposing the top of the bedding to direct sunlight, the vermicompost was gathered from top to bottom using the shade/light technique to remove earthworms from the compost. Due to their sensitivity to light, vermicomposting earthworms hide from direct sunshine. Sieving was also done to separate the earthworms and cocoons.

### 2.4 Treatments and experimental design

Treatments consisted of four levels of vermicompost (0, 4, 8 and 12 t ha^−1^), four levels of potassium (0, 50, 100 and 150 kg K ha^−1^) and two locations [Sodo zuria (location-1) and Boloso sore (location-2) districts of Wolaita zone]. The experiment was laid out in a factorial arrangement of Randomized Complete Block Design (RCBD) with three replications. Treatments were assigned to each plot randomly. A single plot measured 3.6 m × 3.6 m. Planting pattern was 60 by 30 cm resulting in six rows each with 12 plants in each plot. A working distance of 1 m and 1.5 m was left between plots and blocks respectively. Data were collected from plants in the central four rows of each plot, not using plants in the border rows as well as plants at both ends of each row.

### 2.5 Experimental procedures

The land was oxen plowed; large clods broken down, leveled and remnant stalks, undecomposed crop residues, weeds, and other unwanted materials were removed. Vermicompost was applied 30 days before planting by cutting open furrows and incorporating the VC into planting rows at a depth of about 15–20 cm. Potassium fertilizer was banded during planting. Uniform apical vine cuttings of about 45 cm in length were planted in the experimental plots by burying 2/3rd of their lengths into the soil at 45° angle on ridges [49].

The vines were planted on the 10th of June 2018 main rainy season as a rain-fed crop. The land was cultivated using a hand hoe at 4, 8 and 12 weeks after planting and hand weeded as required to reduce weed competition. Harvesting the storage roots was undertaken 150 days after planting. During harvesting, vines were cut to the ground level using a sharp sickle. The roots were carefully gathered once the ridges had been dug down to a depth of 30 cm.

### 2.6 Data collection and measurements

Data were taken from ten randomly selected plants from four central rows. Root length is the total length of roots measured using a ruler. Root diameter is the total diameter of roots. Storage roots, pencil-thick roots, fibrous roots and parts of stem remaining underground were recorded as root fresh mass. The average storage root number per plant was determined by dividing the total number of roots by the number of plants in the sample (ten). Marketable root yield includes a total mass of storage roots that fell in the size category of 100–500 g which are free of diseases and defects [49]. Nonmarketable root yield includes the mass of roots that fall in the size category (<100 g) under-sized, (>500 g) over-sized, diseased and defective roots. Total root yield was determined by weighing all roots from the net plot area. It includes both marketable and nonmarketable root yields. Shoot dry mass includes the dry mass of vine and leaves. It was recorded by cutting the plant aboveground shoots at the soil surface which was determined after first being air-dried and further dried in a ventilated oven at 105 °C for 24 h until a constant mass was obtained. Root dry mass includes the dry mass of underground dry biomass. Root-to-top mass ratio was calculated by dividing the total root dry biomass to the total dry above-ground biomass at the time of maturity. The dry matter content of roots was estimated as the ratio of the mass of dry root of the marketable yield to the fresh mass of the same root mass expressed as a percentage after roots were oven-dried at 105 °C for 24 h.

$$\text{PRDM}=\left(\frac{MDMR}{MFR}\right)\times 100$$

Where; PRDM (%) = Percent Root Dry Matter,

MDMR = Mass of Dry Marketable Roots.

MFMR = Mass of Fresh Marketable Roots.

Harvest index (HI) defined as the ratio of economical parts of the crop to the total biomass. Accordingly, it was determined as the ratio of the dry biomass of marketable root yield to the total dry biomass (above-ground and underground biomass) as described by Ref. [50].

$$HI=\left(\frac{RDM}{TDB}\right)\times 100$$

Where; RDM = Root Dry Mass; TDB = Total Dry Biomass; HI = Harvest Index.

### 2.7 Soil sampling and analysis

Soil samples were randomly taken from the entire experimental field before planting using a soil auger in a zigzag pattern. Thirty soil samples were taken from the topsoil layer to a depth of 20 cm and composited in a bucket [51]. The soil was broken into small crumbs and thoroughly mixed. A 1 kg composite sample was prepared for determining physico-chemical properties.

The soil was air-dried and sieved through a 2 mm sieve. Soil pH was determined from the filtered suspension of the soil-to-water ratio using a glass electrode attached to a digital pH meter [51]. The texture of the soil was determined by the sedimentation method. The soil samples were analyzed for total nitrogen, available phosphorus, exchangeable potassium, and organic carbon. Organic carbon was determined by the method stated by Ref. [52]. Total nitrogen was determined using the Kjeldahl method [53]. Available phosphorus and extractable potassium were estimated through Mehlich 3 method [54]. The chemical properties of the vermicompost were estimated with similar procedures used for the experimental soils.

### 2.8 Data analysis

Data were subjected to analysis of variance (ANOVA) using an appropriate three factor factorial experiment in RCBD according to the General Linear Model (GLM) of the 18th version of GenStat statistical software, and GraphPad Prism version 9.5.1. All significant pairs of treatment means were compared using LSD (Least Significant Difference Test) at a 5 % level of significance. Correlation analyses were performed to detect the linear relationship among parameters.

## 3 Result

The main and interaction effects of VC and K mineral fertilizer significantly influenced yield and yield-related parameters of sweet potato, such as shoot dry mass, root related parameters. None of the parameters has been affected by the interaction effects of VC∗K∗Location.

The combined analysis of mean squares showed that the interaction effects of VC and K (Table 2) significantly affected the number of roots per plant, root length, and root diameter. The number of roots per plant, root length, and root diameter were maximum (6.677, 14.67 cm, and 6.428 cm respectively) after combined application of 12 t VC ha^−1^ and 100 kg K ha^−1^ over not fertilized treatments (Table 4). Similar results of the number of roots per plant and root length were observed from the interaction effect of 12 t VC ha^−1^ and 150 kg K ha^−1^. Maximum root diameter (6.428 cm) was observed from the interaction effect of 12 t VC ha^−1^ and 150 kg K ha^−1^ which was equal to the interaction effect of 8 t VC ha^−1^ and 150 kg K ha^−1^. Root dry mass was influenced by the main and interaction effects of VC and K fertilizer (Table 2). Maximum root dry mass yield was recorded from the combined application of 12 t VC ha^−1^ and 150 kg K ha^−1^ (19.19 t ha^−1^) (Table 6). It was statistically similar to the combined application of 8 t VC ha^−1^ and 150 kg K ha^−1^ and was followed by combined effects of 12 t VC ha^−1^ and 100 kg K ha^−1^ (Table 4). The minimum magnitude of all parameters is recorded from the control plots.

**Table 2:** Mean square values of the effects of VC, K and location (L) on growth and yield parameters of sweet potato in Wolaita zone, southern Ethiopia.

SV	df	RNpp	RL (cm)	RD (cm)	RDMY (t ha^−1^)	SDM (t ha^−1^)
Rep	2	0.4977	3.4050	1.9469	0.248	2.308
VC	3	34.3903∗∗∗	155.1171∗∗∗	27.8310∗∗∗	485.008∗∗∗	420.506∗∗∗
K	3	6.1788∗∗∗	24.6772∗∗∗	12.1253∗∗∗	185.846∗∗∗	128.964∗∗∗
L	1	55.8150∗∗∗	233.4696∗∗∗	9.7729∗∗∗	9.723∗∗∗	15.760∗∗∗
VC × K	9	1.1198∗∗∗	3.0687∗∗∗	0.6469∗∗∗	16.380∗∗∗	4.382∗∗∗
VC × L	3	1.0407∗∗∗	4.5479∗∗∗	1.2613∗∗∗	1.106 ^NS^	11.454∗∗∗
K × L	3	0.2049 ^NS^	0.3798 ^NS^	0.0174 ^NS^	0.605 ^NS^	0.238^NS^
VC × K × L	9	0.1522 ^NS^	0.5418 ^NS^	0.0820 ^NS^	0.205 ^NS^	1.375^NS^
Error	62	0.1716	0.4959	0.1739	1.019	1.229
CV		9.4	6.2	9.2	9.8	8.3

SV source of variation, df degree of freedom, VC vermicompost, L location, SDM shoot dry mass, RNpp total root number per plant, RD root diameter, RDMY root dry mass yield, ∗ Significant at 5 %, ∗∗ significant at 1 %, ∗∗∗ significant at 0.1 %.

**Table 3:** Mean square values of the effects of VC, K and location (L) on growth and yield parameters of sweet potato in Wolaita zone, southern Ethiopia.

SV	df	RTR	MRY (t ha^−1^)	TRY (t ha^−1^)	NMRY (t ha^−1^)	HI
Rep	2	0.002559	12.254	0.780	9.845	0.9252
VC	3	0.433295∗∗∗	1948.044∗∗∗	3402.019∗∗∗	240.752∗∗∗	163.0177∗∗∗
K	3	0.256620∗∗∗	865.639∗∗∗	852.891∗∗∗	2.978^NS^	105.4040∗∗∗
L	1	0.025026∗	351.479∗∗∗	83.272∗∗∗	693.913∗∗∗	3.9539∗
VC × K	9	0.044926∗∗∗	89.820∗∗∗	53.894∗∗∗	10.256∗	9.9563∗∗∗
VC × L	3	0.034509∗∗∗	105.777∗∗∗	6.576 ^NS^	77.393∗∗∗	4.1886∗∗∗
K × L	3	0.006145 ^NS^	35.014∗	8.896 ^NS^	6.331^NS^	0.2397 ^NS^
VC × K × L	9	0.003914 ^NS^	10.402^NS^	2.318 ^NS^	7.251^NS^	0.5018 ^NS^
Error	62	0.04009	9.032	7.220	4.688	0.7436
CV		11.1	11.1	7.3	23.4	8.3

SV source of variation, df degree of freedom, VC vermicompost, L location, RTR root to top ratio, MRY marketable root yield, NRMY nonmarketable root yield, TRY total root yield. ∗ Significant at 5 %, ∗∗ significant at 1 %, ∗∗∗ significant at 0.1 %.

**Table 4:** Interaction effect of VC and K on root growth variables and yields of sweet potato in Wolaita zone, southern Ethiopia.

Treatments		RNP^−1^	RL (cm)	RD (cm)	UMRY (t ha^−1^)	TRY (t ha^−1^)	HI (%)
VC t ha^−1^	K kg ha^−1^						
0	0	2.780 g	7.50 g	2.958 g	6.37 e	21.96 k	6.95 h
	50	2.857 g	8.15 fg	3.157 fg	6.84 e	23.77 jk	7.22 h
	100	2.965 fg	8.89 ef	3.370 fg	7.07 e	26.38 ij	8.01 fgh
	150	3.380 ef	9.34 e	3.557 f	7.78 de	28.70 hi	8.83 efg
4	0	3.797 de	8.51 ef	3.292 fg	7.00 e	24.31 jk	7.33 h
	50	3.963 d	9.18 e	3.548 f	7.16 e	26.23 ij	7.78 gh
	100	4.047 d	10.95 cd	4.603 de	8.56 cde	30.39 h	8.86 ef
	150	4.055 d	11.26 cd	4.842 cd	8.70 cde	33.05 g	9.90 e
8	0	4.030 d	10.93 d	4.158 e	13.73 ab	37.18 f	8.94 ef
	50	4.213 d	11.88 c	4.648 d	13.50 ab	40.35 e	9.89 e
	100	5.713 b	14.37 ab	5.916 b	13.35 ab	49.36 c	13.52 c
	150	5.573 b	14.24 ab	6.179 ab	11.56 bc	56.34 ab	15.94 a
12	0	4.850 c	13.62 b	4.542 de	15.26 a	39.62 ef	9.75 e
	50	5.297 bc	14.04 ab	5.312 c	12.86 ab	43.92 d	11.03 d
	100	6.677 a	14.67 a	6.172 ab	11.89 b	53.81 b	14.80 b
	150	6.297 a	13.61 b	6.428 a	10.90 bcd	57.35 a	16.50 a
SEM		0.1855	0.3337	0.1731	1.127	0.930	0.381
LSD (5 %)		0.5225	0.9402	0.4877	3.176	2.619	1.072
CV (%)		10.3	7.2	9.3	27.2	6.1	9.0

VC vermicompost, SEM standard error of means, CV coefficient of variance. RNP^−1^ root number per plant, RL root length, RD root diameter, RDMY root dry mass yield, MRY marketable root yield, UMRY nonmarketable root yield, TRY total root yield, HI harvest index. Means within a column with similar letters are not statistically different.

The main and interaction effects of VC and mineral K fertilizer (Table 3) significantly influenced total tuberous root yield, and marketable and nonmarketable root yields. Maximum total tuberous root yield (57.35 t ha^−1^) and marketable root yield (46.45 t ha^−1^) were found from the combined application of 12 t VC ha^−1^ and 150 kg K ha^−1^ and both of the results were equivalent to the combined effect of 8 t VC ha^−1^ and 150 kg K ha^−1^ (Table 4, Table 5). Minimum total tuberous root yield (21.96 t ha^−1^) and marketable root yield (15.60 t ha^−1^) were from nil fertilization. Nonmarketable root yield was maximum (15.26 t ha^−1^) from the sole application of 12 t VC ha^−1^ and minimum (6.37 t ha^−1^) from unfertilized ones.

**Table 5:** Interaction effects of VC and K on marketable root yield (t ha^−1^) of sweet potato in two districts of Wolaita zone, southern Ethiopia.

VC	K			
	0	50	100	150
0	15.60 i	16.93 hi	19.31 ghi	20.93 fgh
4	17.31 hi	19.06 ghi	21.84 fg	24.35 ef
8	23.45 ef	26.85 e	35.67 c	44.78 ab
12	26.85 e	31.39 d	41.91 b	46.45 a

SEM	1.448			
LSD (5 %)	4.080			
CV (%)	13.1			

VC vermicompost, SEM standard error of means, CV coefficient of variance. Means within a column with similar letters are not statistically different.

**Table 6:** Interaction effects of VC and K on root dry mass yield (t ha^−1^) of sweet potato in two districts of Wolaita zone, southern Ethiopia.

VC	K			
	0	50	100	150
0	4.64 j	5.25 j	6.29 hi	7.17 gh
4	5.38 ij	6.30 hi	7.95 g	9.11 f
8	9.25 f	10.55 e	15.31 c	18.59 a
12	10.71 e	12.28 d	17.48 b	19.19 a

SEM	0.329			
LSD (5 %)	0.925			
CV (%)	7.8			

VC vermicompost, SEM standard error of means, CV coefficient of variance. RDMC root dry matter content. Means within a column with similar letters are not statistically different.

The interaction effect of VC and mineral K fertilizer was significantly influenced shoot-dry mass (Table 2). Shoot-dry mass was increasing in response to increasing rates of both types of fertilizers. It was maximum (19.36 t ha^−1^) was at the combined application of 12 t VC ha^−1^ and 150 kg ha^−1^ K mineral fertilizer (Table 7). This figure was statistically the same as with combined application of 12 t VC ha^−1^ and 100 kg ha^−1^ K or 8 t VC ha^−1^ and 150 kg ha^−1^ K. Minimum shoot dry mass (6.64 t ha^−1^) was recorded from the control treatments. Root to top ratio (in dry mass basis) expresses the dry matter partitioning between roots, leaves and stems, during growth and development [55]. It was significantly affected by the interaction effect of VC and mineral K fertilizer (Table 3). The maximum root-to-top ratio was recorded from 12 t VC ha^−1^ and 150 kg K ha^−1^ which was the same as 8 t VC ha^−1^ and 150 kg K ha^−1^ (Fig. 3). Harvest index (HI) is the ratio of the economical biomass yield to the total biomass. It was significantly affected by the main and interaction effect of VC and mineral K fertilizer (Table 3). Maximum HI (16.50 %) was obtained from the combined application of 12 t VC ha^−1^ and 150 kg K ha^−1^. It was at par with the combined effect of 8 t VC ha^−1^ and 150 kg K ha^−1^. Minimum HI (6.95 %) was recorded from unfertilized plots. Root-to-top ratio (RTR) significantly affected by the main and interaction effects of VC and K fertilizer. It was maximum after combined application of 8 t VC ha^−1^ and 150 kg K ha^−1^ (Fig. 2) though equivalent to the effect of 12 t VC ha^−1^ and 150 kg K ha^−1^.

**Table 7:** Interaction effect of VC and K on shoot dry mass (t ha^−1^) of sweet potato in two districts of Wolaita zone, southern Ethiopia.

VC	K			
	0	50	100	150
0	6.64 k	7.65 jk	9.16 ij	9.79 hi
4	8.30 ij	10.88 gh	13.26 ef	14.77 de
8	11.91 fg	13.98 de	17.51 bc	18.86 ab
12	15.42 d	17.23 c	19.22 a	19.36 a

SEM	1.337			
LSD (5 %)	1.537			
CV (%)	10.0			

VC vermicompost, SEM standard error of means, CV coefficient of variance. SDM shoot Dry mass. Means within a column with similar letters are not statistically different.

## 4 Discussion

Growth and yield components of sweet potato increased with increasing combined application of VC and K. Soil conditioning with combined application of VC and K mineral fertilizer promoted photo-assimilate formation, partitioning and conversion into dry matter [56]. The number of roots per plant, root length, and root diameter were increased by 6.677, 14.67 cm, and 6.428 cm respectively after combined application of 12 t VC ha^−1^ and 100 kg K ha^−1^ over not fertilized treatments (Table 4). Nonmarketable yield increased from 6.37 to 15.26 t ha^−1^ in response to nil to maximum rate (15.26 t ha^−1^) of sole application of VC respectively. The results are in line with Abd El-Baky et al. (2010) who reported an increased root length, root diameter and mean mass of roots up to 150 kg K ha^−1^. The increment of nonmarketable yield with increasing rates of sole application of VC may indicate that increased rates of more VC might increase the soil N content that might promote growth of roots and initiation of root primordia. Yet the amount of K from VC might not be sufficient for maximum dry matter formation. Storage roots of sweet potatoes are formed after adventitious roots developed from preformed root primordia on the nodes, wound callus on stems or petioles, and lateral roots developed on unthicken adventitious roots, pencil roots, and storage roots [55,57,58]. Thickening of adventitious roots into storage roots occurs in the formation of thin-walled, starch-storing parenchyma cells under favorable conditions [10,55,59]. It requires adequate soil moisture and nutrient supplements for promoting rapid and uniform root development.

Combined application of 12 t VC ha^−1^ with 150 kg ha^−1^ K mineral fertilizer increased shoot dry biomass of sweet potato by 75.8 % shoot dry biomass of sweet potato compared with the control treatments. The increase in root dry mass with increasing combined rates of VC and K might be attributed to the positive influence of VC and K on dry matter formation. Supporting this finding, Kashem et al. (2015) reported a 52 % shoot biomass increase from 20 t VC ha^−1^ on tomatoes. The authors reported that VC stimulated a carpophore formation in Agaricus bisporus. VC contains growth-promoting phytohormones such as gibberellins (GA_3_), cytokinins (IPA), and auxins (IAA) [20,21]. The increment in shoot fresh mass in the current study might be due to the growth-stimulating effects of humic acid, soil microbial activity, and N accessibility in the soil provided by the VC [11,20,55,60]. [61] reported a 25.4 % increase in the dry matter content of sweet potato from the combined application of 20 t VC ha^−1^ and 200 kg K ha^−1^. Storage roots from control treatments were small, succulent and contained more moisture whereas those treated with higher levels of VC and K were larger, firmer and contain low moisture.

Dry matter content in sweet potatoes is directly correlated with starch content [55,62] which needs higher soil organic matter. Starch-synthesizing enzymes have an explicit requirement for K ions. The increase in root dry mass and dry matter content with increasing rates of VC and K might be because VC provides the required amount of soil organic matter, N and maintains the optimum N: K ratio. K also regulates the opening and closing of stomates which in turn improves photosynthesis and photo-assimilation that would accumulate in the form of starch in the storage roots and might increase its dry matter content.

Total tuberous root yield and marketable root yield were increased by 61.7 % and 66.4 % respectively from the combined application of 12 t VC ha^−1^ and 150 kg K ha^−1^ over not fertilized plots (Table 4, Table 5). Both of these results were similar to the combined effects of 8 t VC ha^−1^ and 150 kg K ha^−1^. In contrast, Blouin et al. [63] reported a root yield reduction from the combined use of VC with N mineral fertilizer. The authors justified that increased availability of N from VC and commercial fertilizers might cause excessive vegetative growth at the expense of the economical part during dry matter partitioning and assimilate transportation.

Total roots and marketable yield increment in the current study might be attributed to earthworms' ability to promote the mineralization of the soil's organic matter and litter, and K's role to increase leaf photosynthetic capacity, turgor potential and export of fixed carbon from leaves to storage roots [64]. The translocation of photosynthates from the green parts of the plant (leaf) to the storage root is of utmost importance in the building up of the storage organs (roots). K is involved in the hydration and organization of cell protoplasm thereby maintaining turgor and facilitating dry matter partitioning [32,65]. It includes the formation of assimilates in the green parts of the plant as well as the conversion of assimilates in the storage organs. Nutrient supplements from VC and K fertilizers, as well as the osmotic effect of K^+^ ions, might improve the photosynthesis and translocation processes resulting in increased storage root proportions than tops [65,66].

Young adventitious roots initiated from nodal and inter-nodal regions of an underground vine cutting potentially developed into enlarged storage roots under optimum nutrient supplement. Hence, the combined application of VC and K had a remarkable effect on the marketable and total root yield of sweet potatoes. Lower rates of VC (≤4 t ha^−1^) and K (≤50 kg ha^−1^) resulted in small-sized roots and lower marketable. Most of the storage roots from these treatments were fibrous and pencil roots which agree with Belehu [55] and Truong et al. [10].

**Fig. 1:**
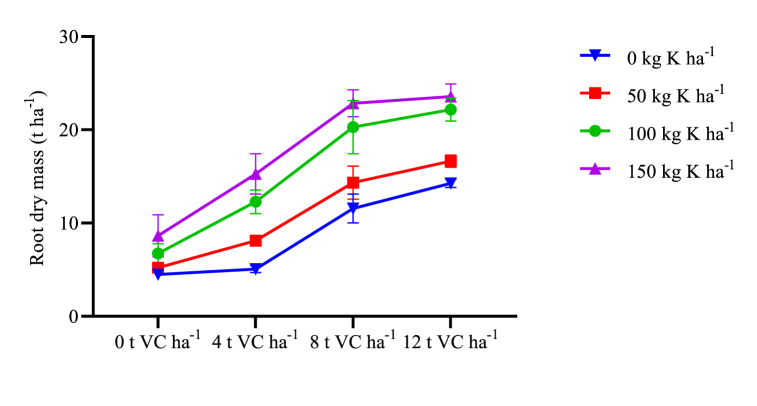
Interaction effects of VC and K mineral fertilizer on root dry mass yield of sweet potato.

**Fig. 2:**
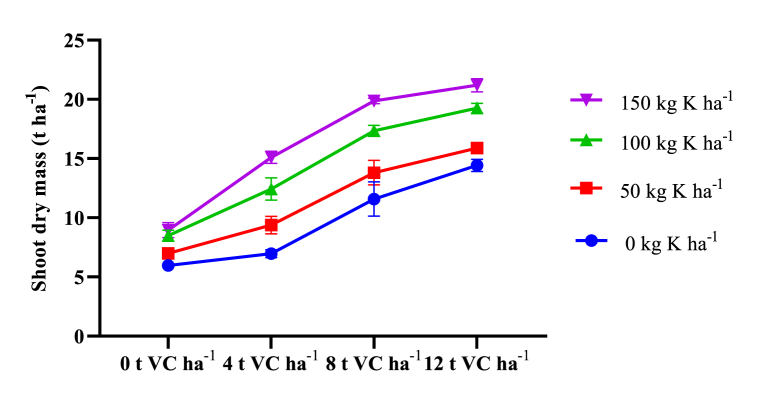
Interaction effects of VC and K mineral fertilizer on shoot dry mass yield of sweet potato.

Combined application of VC and mineral K fertilizer boosted root dry mass and shoot dry mass (Fig. 1, Fig. 2). Lower rates of both fertilizers had lower root dry mass and shoot dry mass. The tuberous roots obtained from the sole application VC, even from maximum rates, were small, lignified, and mostly nonmarketable. This may be because hormone-like substances in vermicompost (VC) could enhance root initiation. Potassium typically works synergistically with other photosynthetically active elements, such as Mg, to facilitatephotosynthesis and photo-assimilating distribution from the vegetative sources to the root sink. This imbalance may lead to an increase in non-marketable yield. Even at the highest rate, the vermicompost alone might not be able to supply the required amount of K to undertake its crucial role during photosynthesis, translocation and storage of assimilates as explained by Refs. [19,21,67]. Gao et al. [68] reported low stomatal conductance and net photosynthetic rate (Pn) accompanied by decreased intercellular CO_2_ concentration from sweet potatoes not supplied with K fertilizer. In line with this finding, Latha et al. [11] noted that soils with an inadequate supply of nutrients favor the lignification of adventitious roots leading to the formation of pencil-like roots and more vine growth. The sole application of VC resulted in maximum unmarketable yield (15.26 t ha^−1^) (Table 4) and excessive growth of aboveground shoots while its integration with K improves the development of storage roots. When K moves into the guard cells around the stomates, the cells accumulate water, swell, increase cell turgor pressure, and enhance root diameter and size [69]. Van Groenigen et a [70]. reported that earthworm casts (VC) did not affect N concentration in the aboveground biomass of grain crops and ryegrass. Hence it can be noted that even though VC increases concentrations and availability of N, P and K nutrients in the soil its combined application with the mineral K fertilizer enhances the root yield of sweet potatoes [71].

**Fig. 3:**
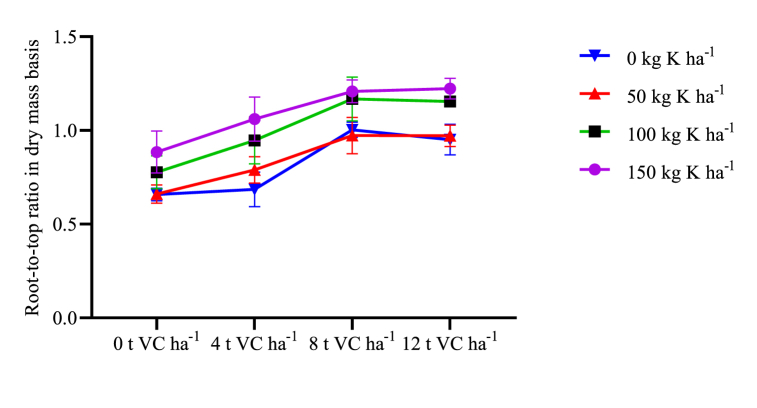
Interaction effects of VC and K mineral fertilizer on dry mass base root-to-top ratio of sweet potato.

Harvest index describes plant's capacity to allocate biomass (assimilates) into the formed reproductive parts. HI was increased by 57.9 % after the combined application of 12 t VC ha^−1^ with 150 kg K ha^−1^ compared with not fertilized plots. Root-to-top ratio (RTR) refers to the dry mass of root biomass divided by the dry mass of shoot biomass. It depends upon the partitioning of photosynthate which may be influenced by environmental stimuli. RTR increased from zero to maximum rates of VC and K. The highest HI and RTR observed with the combined application of higher rates of VC (8 or 12 t ha^−1^) and 150 kg K ha^−1^ may be attributed to the substantial supply of nutrients (N, P, K) from VC, along with its soil conditioning properties. Additionally, potassium facilitates the transport of photosynthates and enhances the photosynthetic efficiency of leaves. Besides, dry matter partitioning to storage roots and other parts of the plant might be promoted [72].

Fortunately, growth and yield related components of sweet potato had a significant and strong positive correlation. RL, RNpp, RD, had strong relationship with TRY (0.9376, 0.9133, 0.9824), MRY (0.8671, 0.7232, 0.7353), RDM (0.9190, 0.9118, 0.9688), SDM (0.9185, 0.8987, 0.9557), RTR (0.7947, 0.7531, 0.8219) and HI (0.7156, 0.7140, 0.7533) (Table 8). Nonmarketable yield had also a significant and strong relationship with RL (0.8179), RNpp (0.7232), RD (0.7353), MRY (0.6842), RDM (0.7800), SDM (0.7732), RTR (0.7375) and HI (0.7113). It may imply that combined application of VC and K mineral fertilizer could improve both the shoot and root of sweet potato. Dry matter partitioning between the aboveground and underground biomass might have been balanced. Root dry mass yield and shoot dry mass were positively and strongly correlated (>0.9) with each other. Overall, most growth and root yield traits show moderate to strong correlations.

**Table 8:** Correlation between growth and root yield traits of sweet potato as influenced by combined application of VC and K in Wolaita zone, southern Ethiopia.

RL (cm)	1	–									
RD (cm)	2	0.9063∗∗∗	–								
RNpp	3	0.9008∗∗∗	0.9018∗∗∗	–							
TRY (t ha^−1^)	4	0.9376∗∗∗	0.9590∗∗∗	0.9133∗∗∗	–						
MRY (t ha^−1^)	5	0.8671∗∗∗	0.9388∗∗∗	0.9100∗∗∗	0.9385∗∗∗	–					
NMRY (t ha^−1^)	6	0.8179∗∗∗	0.7353∗∗∗	0.7232∗∗∗	0.8358∗∗∗	0.6842∗∗∗	–				
RDM (t ha^−1^)	7	0.9190∗∗∗	0.9688∗∗∗	0.9118∗∗∗	0.9824∗∗∗	0.9537∗∗∗	0.7800∗∗∗	–			
SDM (t ha^−1^)	8	0.9185∗∗∗	0.9557∗∗∗	0.8987∗∗∗	0.9654∗∗∗	0.9384∗∗∗	0.7732∗∗∗	0.9752∗∗∗	–		
RTR	9	0.7947∗∗∗	0.8219∗∗∗	0.7531∗∗∗	0.8548∗∗∗	0.7590∗∗∗	0.7375∗∗∗	0.8622∗∗∗	0.7513∗∗∗	–	
HI (%)	10	0.7156∗∗∗	0.7533∗∗∗	0.7140∗∗∗	0.7719∗∗∗	0.6567∗∗∗	0.7113∗∗∗	0.7766∗∗∗	0.7004∗∗∗	0.8781∗∗∗	–
		1	2	3	4	5	6	7	8	9	10

RNP^−1^ root number per plant, RL root length, RD root diameter, RDM root dry mass, SDM shoot dry mass, RTR root to top ratio, MRY marketable root yield, NMRY nonmarketable root yield, TRY total root yield, HI harvest index. ∗∗∗ significant at 0.1 %.

## 5 Conclusion

Generally, growth and root yield were increasing with increasing rates of both fertilizers. Experimental plots that received lower rates of VC (≤4 t ha^−1^) and K (≤50 kg ha^−1^) had an inferior shoot and root development to ≥4 t ha^−1^) and ≥50 kg ha^−1^ respectively. Combined application of 12 or 8 t VC ha^−1^ with 150 kg K ha^−1^ brought maximum root dry mass (19.19 t ha^−1^), shoot dry mass (19.36 t ha^−1^), marketable root yield (46.45 t ha^−1^), root-to-top ratio and HI (16.50 %). Equivalent maximum results obtained from combined effects of 8 t VC ha^−1^ and 150 kg K ha^−1^. Hence, sweet potato producers in the study area can use a combined application of 8 t VC ha^−1^ and 150 kg K ha^−1^ to maximize sweet potato root yield as well as aboveground biomass. Future research is suggested on economic analysis of the combined use of VC and mineral K fertilizers in different locations.

## Code availability (software application or custom code)

Not applicable.

## CRediT authorship contribution statement

**Alemu Degwale:** Writing – original draft, Supervision, Resources, Project administration, Methodology, Investigation, Funding acquisition, Formal analysis, Conceptualization. **Geletaw Kebede:** Writing – review & editing, Writing – original draft, Visualization, Methodology, Data curation. **Ashenafi Woldeselassie:** Writing – review & editing, Validation, Resources, Funding acquisition, Data curation, Conceptualization. **Fanuel Laekemariam:** Writing – original draft, Visualization, Validation, Investigation, Funding acquisition.

## Ethics approval and consent to participate

Not Applicable.

## Consent for publication

Not Applicable.

## Availability of data and materials

The authors of this manuscript would like to confirm that, all the data will be available from the corresponding author with a reasonable request.

## Funding

This research work was funded by Wolaita Sodo University, Ethiopia.

## Declaration of competing interest

The authors declare the following financial interests/personal relationships which may be considered as potential competing interests:Alemu Degwale reports equipment, drugs, or supplies was provided by Amhara Regional Agricultural Research Institute. If there are other authors, they declare that they have no known competing financial interests or personal relationships that could have appeared to influence the work reported in this paper.
